# Melatonin: A Multifunctional Factor in Plants

**DOI:** 10.3390/ijms19051528

**Published:** 2018-05-21

**Authors:** Jibiao Fan, Yan Xie, Zaichao Zhang, Liang Chen

**Affiliations:** 1 College of Animal Science and Technology, Yangzhou University, Yangzhou 225009, China; 006298@yzu.edu.cn; 2 Key Laboratory of Plant Germplasm Enhancement and Specialty Agriculture, Wuhan Botanical Garden, Chinese Academy of Sciences, Wuhan 430074, China; xieyan@wbgcas.cn; 3 Jiangsu Key Laboratory for the Chemistry of Low-Dimensional Materials, School of Chemistry and Chemical Engineering, Huaiyin Normal University, Huai’an 223300, China; zhangzc@hytc.edu.cn

**Keywords:** melatonin, plant, biosynthesis, stress tolerance

## Abstract

Melatonin (*N*-acetyl-5-methoxy-tryptamine) is a universal molecule that is present in animals and plants. It has been detected in different kinds of plants and organs in different levels. Melatonin in plants shares the same initial biosynthesis compound with auxin, and therefore functions as indole-3-acetic acid like hormones. Moreover, melatonin is involved in regulating plant growth and development, protecting plants against biotic and abiotic stresses, such as salt, drought, cold, heat and heavy metal stresses. Melatonin improves the stress tolerance of plants via a direct pathway, which scavenges reactive oxygen species directly, and indirect pathways, such as increasing antioxidate enzymes activity, photosynthetic efficiency and metabolites content. In addition, melatonin plays a role in regulating gene expression, and hence affects performance of plants. In this review, the biosynthesis pathway, growth and development regulation, and the environment stress response of melatonin in plants are summarized and future research directions and priorities of melatonin in plants are speculated.

## 1. Introduction

In the growth and development process of plants, numerous factors, especially environmental stresses, are involved in molding the status of the plant. Biotic and abiotic stresses will induce yield reduction, growth retardation, senescence and even death. Plants develop various strategies to alleviate the damage induced by different stresses. Plenty of molecules such as ethylene [1], nitric oxide [2], hydrogen sulfide [3], calcium [4] and phytohormones, such as jasmonic acid [5], gibberellin acid [6] and abscisic acid [7], are involved in plant stress responses. Nowadays, another amazing molecule named melatonin draws the attention of researchers. Plenty of investigations found that melatonin plays an essential role in plant development and stress responses.

Melatonin (*N*-acetyl-5-methoxy-tryptamine) was so-named, when it was first identified in 1958, because it could reverse the darkening effect of melanocyte stimulating hormone (MSH) [8]. In the first four decades after melatonin was isolated, studies on melatonin focused on animals. It was shown that melatonin played key roles in the regulation of antioxidant enzymes activity [9,10], circadian rhythms [11], coronary heart disease [12], Alzheimer’s disease [13], physical condition and emotional status [14]. In 1995, melatonin was discovered in plants as well [15,16]. Since then, melatonin has been detected in different plant species (Table 1). Further investigations have found that melatonin is also a widespread and multifunctional metabolite in the plant kingdom. It is distributed in the leaves, stems, roots, fruits and seeds of various plants (Table 1). It is not only associated with plant stress response, such as cold [17], salinity [18], drought [19], oxidative [20] and nutritional deficiency [21], but also relative to development regulation, such as growth [22], senescence [23,24], root organogenesis [25] and flowering [26].

Recently, the regulation and function of melatonin in plants has been extensively and deeply investigated, and regulation mechanisms related to melatonin have increasingly been discovered. In this review, the biosynthesis pathway, growth and development regulation, and environment stress response of melatonin in plants are summarized and future research directions and priorities into melatonin in plants are speculated. It will contribute to the understanding of the current situation and consider the future direction of melatonin in plants research.

## 2. Biosynthesis of Melatonin in Plant

**Figure 1:**
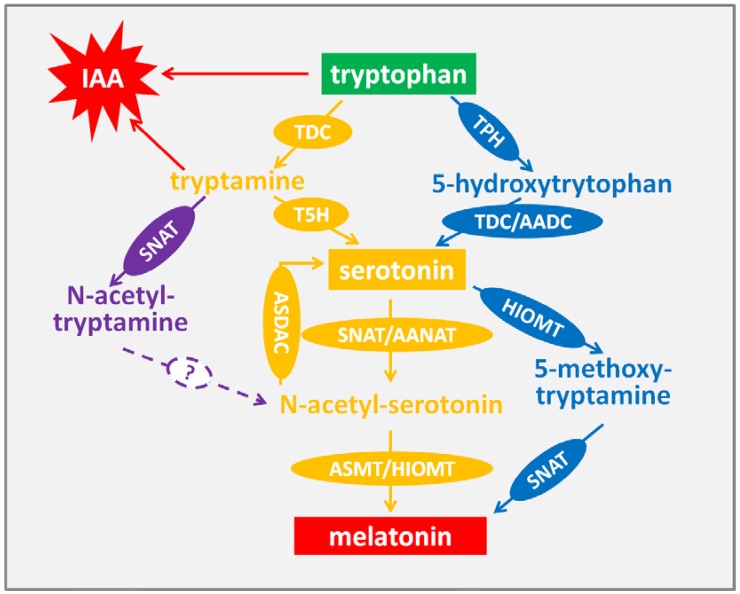
Proposed biosynthesis pathway of melatonin in plants. TDC, Tryptophan decarboxylase; T5H, tryptamine 5-hydroxylase; SNAT: serotonin-*N*-acetyltransferase; AANAT: arylalkylamine *N*-acetyltransferase; ASMT: *N*-aceylserotonin methyltransferase; HIOMT, hydroxyindole-*O*-methyltransferase; AADC, aromatic-l-amino-acid decarboxylase; TPH: tryptophan hydroxylase; ASDAC: *N*-acetylserotonin deacetylase.

**Figure 2:**
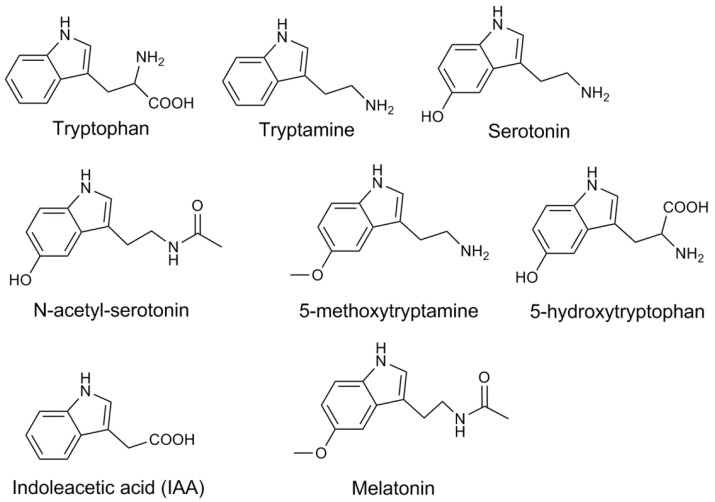
Chemical structures of all the compounds in melatonin biosynthesis pathway.

In the animal system, melatonin is secreted in the pineal gland. However, there is no such organ in plants, and this difference implies that the melatonin biosynthesis pathway in plants is slightly different from that in animals. Contrary to the animal system, melatonin distributes in various organs, such as the root, stem and leaf in plants. Multiple factors can stimulate melatonin biosynthesis in plants. Light is one environment factor that regulates the biosynthesis of melatonin [27]. In addition, the development process, such as fruit maturation [28], leaf development [29] and senescence [30], and environment stresses, including ultraviolet-B (UV-B) radiation [31], drought, cold [32] and heat [33], are involved to stimulate biosynthesis of melatonin. In a wide range of plant species, melatonin biosynthesis begins from tryptophan. It is catalyzed by tryptophan decarboxylase (TDC) and converted to tryptamine, and then tryptamine 5-hydroxylase (T5H) catalyzes tryptamine to serotonin, which will be converted to melatonin via two steps [34]. In some other plants, such as *Hypericum perforatum*, tryptophan is catalyzed into 5-hydroxytrytophan by tryptophan 5-hydroxylase (TPH), and then TDC/AADC (aromatic-l-amino-acid decarboxylase) converts 5-hydroxytrytophan to serotonin [35]. This pathway is the same as that in animals. In the next two steps, serotonin is converted to *N*-acetyl-serotonin by serotonin *N*-acetyltransferase (SNAT)/arylalkylamine *N*-acetyltransferase (AANAT), and then *N*-acetyl-serotonin methyltransferase (ASMT)/hydroxyindole-*O*-methyltransferase (HIOMT) catalyzes *N*-acetyl-serotonin into melatonin. Additionally, tryptamine can be catalyzed by SNAT into *N*-acetyl-tryptamine, which is not further converted into *N*-acetyl-serotonin by T5H [36]. It is difficult to determine whether there is a pathway to converting *N*-acetyl-tryptamine into *N*-acetyl-serotonin. The other pathway is to convert serotonin into 5-methoxy-tryptamine by HIOMT and, finally, to catalyze 5-methoxy-tryptamine into melatonin by SNAT [37,38] (Figure 1). Meanwhile, the chemical structures of the compounds are showed in Figure 2. Recently, a reverse melatonin pathway was reported, in which *N*-acetyl-serotonin is converted into serotonin by *N*-acetyl-serotonin deacetylase [39]. Furthermore, tryptophan is not only the resource of melatonin, but also the precursor of indole-3-acetic acid (IAA), maybe implying the multifunctional role of melatonin in plants.

## 3. Endogenous Melatonin in Plants

Since melatonin was identified in plants [15,16], the endogenous melatonin in plants was investigated deeply. It was proved that melatonin widely existed in various plant species, including medicinal herbs, crops and fruit, although melatonin content was found to vary in different plants (see in Table 1). For example, the melatonin content was 1000 ng/g in several herbs, which was 15–100 fold higher than the average level in other plants [40,41,42]. In addition, the detection of lupin (*Lupinus albus* L.) and barley (*Hordeum Vulgare* L.) showed that distributions of endogenous melatonin were in conformity in different organs and development periods [43]. Similar results were also found in morning glory (*Pharbitis nil* Choisy) and tomato (*Lycopersicon esculentum* Mill.) plants: melatonin content generally increased during the ripening process [28]. Investigation into rice (*Oryza sativa* cv. Asahi) leaves in the senescence process demonstrated that melatonin levels were higher in leaves under constant light conditions than under constant darkness conditions. This result suggested that melatonin biosynthesis during senescence development was regulated by light signals [27]. Recently, data showed that endogenous melatonin in plants could be affected by environmental conditions. Melatonin content in tomato plants cultured in open fields was higher than that of plants cultured in chambers [44]. Coincidentally, another research found that the melatonin level of *Vitis vinifera* decreased dramatically in the daytime and was highest in the darkness [45]. These results suggested that light was a suppression factor of melatonin. However, after treated with chemical stress, melatonin content in barley (*Hordeum vulgare* L.) increased significantly [46]. Similar results were detected in lupin (*Lupinus albus*). The contents of endogenous melatonin increased under abiotic stresses [32]. Meanwhile, melatonin in rice seedlings was enhanced by high temperature [33]. All data above suggested that the stress condition could induce the biosynthesis of endogenous melatonin in plants. Additionally, it is proved that melatonin shared the same pathway with indole 3-acetic acid, which is the auxin (Figure 1). *OsIDO* (*Oryza sativa indoleamine 2,3-dioxygenase*) transgenic tomato plant showed lower melatonin content. This suggested that indoleamine 2, 3-dioxygenase (IDO) was involved in the regulation of plant melatonin metabolism [47].

## 4. Functions of Exogenous Melatonin Treatment in Plants

### 4.1. Effect of Melatonin on Plant Drought/Water Tolerance

Drought is a severe stress to plants, especially in agricultural production. Therefore, it is essential to find a phytohormone that could be applied to alleviate drought damages. According to recent studies, melatonin could be used to improve the drought tolerance of plants. In apple plants (*Malus domestica* Borkh.), positive results were detected in oxidative damage, photosynthesis efficiency and senescence progress after 100 μM melatonin treatment under drought conditions [50]. Moreover, in other *Malus* species, the drought tolerance was also improved by melatonin in both drought-tolerant *M. prunifolia* and drought-sensitive *M. hupehensis* plants. Meanwhile, it has also been reported that the abscisic acid (ABA) synthesis gene *MdNCED3* was down-regulated and the catabolic genes, including *MdCYP707A1* and *MdCYP707A2*, were up-regulated. In addition, the plants could regulate the water balance by regulating the expression of related genes. Melatonin was also involved in scavenging H_2_O_2_ and increasing the activity of antioxidant enzymes under drought conditions [51]. Meanwhile, the overexpression of the melatonin synthesis gene, *MzASMT1*, significantly increased the drought tolerance of the transgenic *Arabidopsis* plants [36]. In grapes as well, injuries induced by drought stress, including internal lamellar system of chloroplasts, photosystem efficiency and antioxidant enzyme activity, were alleviated after melatonin treatment [52].

Melatonin not only plays an essential role in improving tolerance to drought, which is a water deficiency stress, but also in increasing tolerance to water stress in plants. The investigation of cucumber (*Cucumis sativus*) under water stress showed that melatonin could stimulate root generation, increase antioxidant enzymes activity and photosynthesis efficiency, and hence the tolerance of water stress was improved [53].

### 4.2. Effect of Melatonin on Plant Cold/Heat Tolerance

Temperature is an important environmental factor. Severe climates, including low and high temperature, can inhibit the growth and development processes of plants. Finding an efficient phytohormone which can be applied to increase the tolerance to extreme temperature of the plant is important in crop breeding. Recently, melatonin was proved to be a candidate hormone that could increase cold and heat stress of the plant. It was reported that, in carrot suspension cells (*Daucus carota* L.), apoptosis induced by 3 °C cold stress was alleviated by melatonin. However, melatonin did not scavenge the reactive oxygen species (ROS) directly. It induced the increase of putrescine and spermidine levels. These interesting results implied that melatonin alleviated cold damage in plants via the regulation of polyamines content [54]. In cucumber (*Cucumis sativus*) seeds, the germination rate dramatically increased from 4% (control) to 83% at 10 °C after application of melatonin [55]. For elite endangered germplasm preservation, cryopreservation method was usually used. In the process of cryopreservation, the plant callus was treated with an extremely low temperature, which induced severe injury to the callus. It was reported that the survival rate of *Rhodiola crenulata* increased significantly in the callus that was pretreated with melatonin [56]. Meanwhile, the shoot tips and dormant winter buds of American elm produced almost 100% regrowth after melatonin pretreatment [57]. This discovery could provide a potential application in the cryopreservation process. Melatonin-pretreated wheat seedlings exhibited higher antioxidant enzymes activity and osmoprotectants levels, suggesting that melatonin can improve the cold resistance of the plant by scavenging ROS and modulating redox balance [58]. Additionally, in bermudagrass (*Cynodon dactylon*), various metabolite concentrations were changed and the photosystem II was improved by exogenous melatonin. Therefore, the cold tolerance of the plant was significantly improved [59,60,61].

Like that of cold stress, melatonin can also be applied to alleviate the heat stress damage of plants. When the plant was exposed to heat stress, the level of melatonin in plant cells increased dramatically [33,62]. In addition, melatonin application could increase heat stress tolerance of the plant. Germination percentages of *Phacelia tanacetifolia* and *Arabidopsis thaliana* seeds were significantly increased by exogenous melatonin [63,64].

### 4.3. Effect of Melatonin on Plant Salt Tolerance

Salt stress is one of the greatest challenges that limits the growth and development of plants across the world. Salt stress induces water deficit and physiological damages to the plant. Meanwhile, plants develop several strategies, such as increasing activity of related enzymes, improving photosynthetic efficiency and regulating gene expressions to tolerate the stress [65]. In recent years, melatonin was reported to play a role in increasing the salt tolerance of plants. In *Malus hupehensis*, growth inhabitation and photosynthetic capacity were improved by application of 0.1 μM melatonin. In addition, exogenous melatonin significantly suppressed the H_2_O_2_ content, and activities of oxidate enzymes, such as ascorbate peroxidase (APX), catalase (CAT) and peroxidase (POD), were enhanced. The results suggested that exogenous melatonin was involved in alleviation of salinity-induced stress in plants [18]. A clearly recognizable effect in the root system of cucumber (*Cucumis sativus* L.) under the salinity condition was detected after melatonin treatment. The density of lateral roots was higher than that of the control, suggesting the positive effect of melatonin on formation of lateral roots under salinity stress [66]. Moreover, exogenous melatonin could increase the salt tolerance and regulate related genes expressions in soybean [67]. Previous studies revealed that phytohormones, such as gibberellic (GA) and abscisic acids (ABA), were involved in improving the salt tolerance of plants [68,69]. Additionally, melatonin was reported to be involved in regulating expressions of the genes that related to biosynthesis and catabolism of GA and ABA, and hence the salt tolerance of cucumber (*Cucumis sativus*) was increased [70].

### 4.4. Effect of Melatonin on Plant Heavy Metal Tolerance

Heavy metal contaminant is a serious environmental problem to all kinds of creatures and especially to plants. However, plants that were treated with melatonin had stronger tolerance to heavy metal stresses according to recent reports. Pea plants (*Pisum sativum*) pretreated with melatonin survived after 100 μM copper treatment, but control plants died. This result suggested that melatonin could enhance the copper tolerance of plants. Since melatonin is safe to animals and humans, as well as inexpensive, it may be a feasible and cost-effective approach to clean environmental contaminations [71]. In algae, the dosage of heavy metals, such as Cd, Pb and Zn, had a positive effect on melatonin levels, and Cd stress tolerance of algae was increased by exogenous melatonin [62]. Seed germination and seedling growth under copper stress were improved after 1 and 10 μM melatonin treatment, while a relatively high concentration of melatonin (100 μM) had a negative effect, which means that it enhanced the toxic effect of copper [72]. Melatonin enhanced the tolerance of Cd stress in *Solanum lycopersicum* by improving the plant growth, photosynthesis and antioxidant enzymes. In addition, the oxidative damage was alleviated after melatonin application. These results suggested that melatonin played multiple roles in protecting plants against Cd stress [73].

## 5. Regulatory Genes Involved in Melatonin Reaction Pathway

Byeon et al. discovered that serotonin *N*-acetyltransferase (SNAT) and *N*-acetylserotonin methyltransferase (ASMT), which are the final two enzymes of the endogenous melatonin biosynthesis pathway in plants, achieved the highest catalytic efficiency values at 55 °C. These results can perhaps explain the dramatic increase of melatonin content in plants after high temperature treatment [33]. The assay of SNAT activity in rice showed that it was inhibited by high serotonin concentration, and, unlike *ASMT*, *SNAT* was expressed constitutively, suggesting that ASMT was the rate-limiting enzyme in plant melatonin biosynthesis [74]. In *Arabidopsis thaliana*, expression of cold- and drought-responsive genes, *COR15a*, *CAMTA1*, ZAT6, *ZAT10* and *ZAT12*, were up-regulated by melatonin, and hence cold tolerance was increased [17,75]. In *Malus domestica*, the transcript levels of a key chlorophyll degradation gene, *pheide a oxygenase* (*PAO*) and, moreover, the *senescence-associated gene 12* (*SAG12*) and *AUXIN RESISTANT 3* (*AXR3*)/*INDOLE-3-ACETIC ACID INDUCIBLE 17* (*IAA17*) were suppressed by melatonin. Therefore, the leaf senescence was delayed [23,30]. Hence, the leaf senescence of the plant was regulated via a melatonin-mediated pathway. In *Malus hupehensis*, ion-channel genes, including *MdNHX1* and *MdAKT1*, and vacuolar Na^+^/H^+^ antiporter, which were critical for promoting the accumulation of Na^+^ or K^+^ inside the vacuoles, were significantly up-regulated under the salinity condition, thereby alleviating salinity-induced damage [18]. The results of RNA-seq in cucumber (*Cucumis sativus*) roots under NaCl stress showed that 77 differentially-expressed genes were regulated by melatonin, and transcription factors, including MYB, WRKY, NAC, ERF, were identified [66,76]. Heat stress response regulator class A1 heat-shock factors (HSFA1s) were significantly up-regulated by exogenous melatonin, and hence heat tolerance of *Arabidopsis* was improved [77].

## 6. Effect of Melatonin on Plant Growth and Development

In the growth and development process of plants, various phytohormones were involved, especially the auxin. As a kind of indoleamine, melatonin shared the same initial compound, which is tryptophan with IAA, so melatonin should play a role in the regulation of growth and development in plants. Melatonin is regarded as a growth-promoting molecule, just as auxin, in lupin hypocotyls, as well as in monocot species, such as canary grass, wheat, barley and oat [78,79], and dicot species, such as Arabidopsis [80], and so it is an auxinic hormone in plants. It was reported that the concentration of melatonin in *Chenopodium rubrum* changed regularly during 12 h light/12 h day cycle, suggesting that melatonin plays a role in circadian rhythms regulation in plants [81]. Flowering of *Chenopodium rubrum* L. seedlings exposed to a single inductive 12 h darkness reduced significantly after the application of 100 and 500 μM melatonin, while no significant change was detected in photoperiodic time. This discovery implied that exogenous melatonin could affect flower development in the early stage of the photoperiod [26]. The leaf size, plant height, pod and seed numbers of soybean plants increased significantly after melatonin treatment, suggesting that exogenous melatonin could improve the growth and seed production of soybean plants [67]. Chlorophyll degradation of barley leaves was slowed down when they were treated with melatonin, revealing the protective role of melatonin in the senescence process of plants [24]. In detached apple (*Malus domestica*) leaves, reduction of chlorophyll content and photosystem efficiency (Fv/Fm) were delayed after melatonin treatment, suggesting that the dark-induced senescence process was delayed by 10 mM melatonin solution [23]. It was shown that H_2_O_2_ accumulation was inhibited and ascorbate peroxidase (APX) activity was enhanced. Simultaneously, melatonin led to higher ascorbic acid (AsA) and glutathione (GSH) contents, but lower dehydroascorbate (DHA) and oxidized glutathione (GSSG), than the control, suggesting that melatonin regulated the plant senescence via an ascorbate-glutathione cycle [23]. Zhang et al. reported that melatonin showed a positive effect on lateral root formation in cucumber plants (*Cucumis sativus*) [66]. Similar to the function of IAA, melatonin could stimulate the expansion of etiolated cotyledons of lupin (*Lupinus albus* L.) [82], suggestive of a possible involvement of melatonin.

In addition, different concentrations of melatonin showed different effects in plants. In *Arabidopsis* seedlings, low melatonin content (10–20 μM) had no significant effect on root length. On the contrary, high melatonin content (200–400 μM) fresh weight was significantly inhibited, and the moderate condition (40 μM) was the optimal condition for the promoted growth and development of the plant [17].

## 7. Effect of Melatonin on Disease Resistance

Plant diseases induced by virus, fungus and bacteria are usually infectious, and even lethal, so it is a severe threat for plants. Therefore, finding a strategy to improve the disease resistance of plants is a key focus in plant breeding. Recently, many positive functions were reported in melatonin-treated plants. Yin et al. reported that resistance of apple plants (*Malus prunifolia*) to Marssonina apple blotch (*Diplocarpon mali*) was improved when pretreated with melatonin. It is shown that the photosystem efficiency, antioxidant and plant defense-related enzymes activity were increased after melatonin treatment [83]. Considering that melatonin is an environmentally-friendly compound, melatonin could be an economical strategy to protect plants against pathogen infections. Melatonin may also be a defense-signaling molecule that plays a role in defense against *Pseudomonas syringae* (*Pst*) DC3000, which is a virulent bacterium in *Arabidopsis* [84]. Further research revealed that both melatonin and nitric oxide (NO) levels were significantly induced by *Pst* DC3000, and there was no significant effect of innate immunity in NO-deficient mutants after melatonin treatment. These results indicated that melatonin improved disease resistance against bacterial pathogen infection via inducing NO production [85]. It was reported that exogenous melatonin could improve the *Fusarium wilt* resistance of banana plants via regulating the expression of *MaHSP90s* [86].

## 8. Conclusions and Future Perspectives

In the last several years, remarkable development on melatonin research in plants has been made. The progress extends the knowledge of melatonin presence, metabolism and functions in plants. As summarized above, melatonin presents in many kinds of plants and organs, while the precise concentrations in different plants and organs are not stable. Since there is no pineal gland in plants, the biosynthesis pathway of melatonin is different in plants from that in animals. Coincidently, the melatonin biosynthesis pathway is homologous with that of auxin in plants, despite some distinguishing enzymes. To date, a mass of studies showed that melatonin played essential roles in improving abiotic and biotic stress tolerance of plants. Concentrations of endogenous melatonin increased in plants under different stress conditions, implying that melatonin was involved in regulating the stress tolerance of plant species. However, some aspects of melatonin in plants, including the metabolism and regulation pathway under stressful conditions, are still unclear.

To understand the metabolism pathway in plants, measuring the concentrations of melatonin in different plants and organs is necessary. Nevertheless, melatonin concentration changes dramatically in the detected plants and organs. Even in the same plant, melatonin content is shifty in different development periods [48]. Yet, how does the melatonin content change from an extremely low level to a high level? Which receptor or protein is related to melatonin content change? These questions are still confusing; molecules or enzymes involved in melatonin regulation are not sufficiently documented. Hence, the signaling mechanisms that regulate the change of melatonin content are still unknown. Generally, melatonin content is very low in leaves and is relatively high in roots and seeds [28,87]. As reported, melatonin is a growth regulator at a low concentration and it preserves the viability of the seed at a high concentration in *Arabidopsis thaliana* [64]. However, the mechanisms of different melatonin distribution in shoots and roots, as well as the functions of melatonin in the organs, still need to be investigated.

Melatonin is a multifunctional factor in plant stress resistance, growth and development process. It is not only a scavenger to reduce reactive oxygen species (ROS), including hydrogen peroxide (H_2_O_2_), superoxide anion (O_2_^−^) and hydroxyl radical (•OH) directly, but also a regulation factor to increase activities of antioxidant enzymes [88,89], metabolite contents [59] and photosynthetic efficiency [60]. In addition, melatonin is involved in NO and ABA pathways [68,82,90], and the related genes expression regulation [91]. Further research should focus on clarifying how melatonin functions as an integrated factor.

Signaling pathways of phytohormones, such as ABA, IAA and GA were investigated well. Therefore, the second messengers and signal transduction involved in modulation reaction and gene expression regulation were clarified. The biosynthesis pathway of melatonin in plants was discovered, and some melatonin-related genes expressions were reported by transcriptomic analysis [59], although the distinct signaling pathway of melatonin still remained unknown. Hence, the signaling pathway of melatonin should be clearly elucidated in further research.

## Figures and Tables

**Table 1:** Distribution of melatonin in plants and organs.

Plant Species	Organ	Ref.
*Lycopersicon pimpinellifolium*	fruit	[16]
*Lycopersicon esculentum* Mill.	fruit	[16]
*Musa nana* Lour.	fruit	[16]
*Cucumis sativus* L.	root	[16]
*Beta vulgaris* L.	leaf	[16]
*Nicotiana tabacum*	stem tuber	[16]
*Solanum tuberosum* L.	hypocotyl	[22]
*Lupinus albus* L.	stem	[25]
*Hypericum perforatum* cv. Anthos	seed	[28]
*Pharbitis nil Choisy*	fruit	[28]
*L. esculen-tum* Mill.	root, leaf, stem, seed	[31]
*Glycyrrhiza uralensis*	fruit	[40]
*Musa ensete*	fruit	[40]
*Fragaria magna*	root	[40]
*Raphnus sativus*	seed	[40]
*Punica granatum*	leaf	[40]
*Brassica oleraceae* var. capitata	leaf	[40]
*Brassica oleraceae* var. botrytis	leaf	[40]
*Brassica rapa*	corm	[40]
*Allium cepa*	corm	[40]
*Allium sativum*	seed	[40]
*Hordeum vulagare*	fruit	[40]
*Ananas comosus*	seed	[40]
*Oryza sativum*	seed	[40]
*Zea mays*	fruit	[40]
*Malus domestica*	tuber	[40]
*Zingiber officinale*	root	[40]
*Daucus carota*	fruit	[40]
*Lycopersicon esculentum*	fruit	[40]
*Cucumis sativus*	cortex	[41]
*Phellodendron amurense* Rupr.	cortex	[41]
*Mori Albae*	leaf	[41]
*Epimedium brevicornum* Maxim	whole leaf	[41]
*Coptis chinensis* Franch	root	[41]
*Rheum palmatum* L.	root	[41]
*Polygala tenuifolia* Willd.	fruit	[41]
*Coruns officinalis* Sieb.	root	[41]
*Gentiana scabra* Bge	whole plant	[41]
*Pirola decorata* H.	root	[41]
*Angelica sinensis* Oliv.	stem	[41]
*Taxillus chinensis* DC	fruit	[41]
*Lycium barbarum* L.	leaf	[41]
*Aloe vela* L.	shoot	[41]
*Andrographis paniculats* Burm.	cortex	[41]
*Eucommia ulmoides* Oliv	seed	[41]
*Raphanus sativus* L.	flower	[41]
*Syzygium aromaticum* L.	fruit	[41]
*Rubus chingii* Hu	root	[41]
*Scrophularia ningpoensis* Hemsl.	shoot	[41]
*Agastaches rugosa*	whole plant	[41]
*Lobelia chinesis* Lour	fruit, seed	[41]
*Ziziphus jujuba* Mill.	root	[41]
*Ophiopogon japonicus*	root	[41]
*Sophora flavescens* Ait.	root	[41]
*Salvia miltiorrhiza* Bge.	root	[41]
*Gentiana macrophylla* Pall.	root	[41]
*Scutellaria amoena* C.H. Wright	whole plant	[41]
*Desmodium styracifolium* Merr.	root	[41]
*Panax notoginsneg* Burk	whole plant	[41]
*Leonurus japonicus* Houtt.	flower	[41]
*Dendranthema morifolium*	root	[41]
*Arnebia euchroma*	root	[41]
*Pueraria lobata* Willd	stem	[41]
*Caulis Polygonam multiflorum* Thunb	flower	[41]
*Lonicera japonica* Thunb	rhizome	[41]
*Curcuma aeruginosa* Roxb	root	[41]
*Glycyrrhiza uralensis* Fisch	root	[41]
*Rehmannia glutinosa*	fruit	[41]
*Schisondra chinensis*	shoot	[41]
*Artemisia annua* L.	root	[41]
*Isatis indigotica* Fort	root	[41]
*Saposhmikovia divaricata*	whole plant	[41]
*Mahonia bealei* (Fort.)	leaf	[41]
*Forsythia suspensa* (Thunb.)	rhizome	[41]
*Polygonatum sibiricum* Delar	leaf	[41]
*Lophartherum gracile* Brongn.	fruit cluster	[41]
*Prunella vulgaris* L.	whole plant	[41]
*Herba Patriniae scabiosaefoliae*	root	[41]
*Angelica biserrata*	stem	[41]
*Cistanche desericola* Y.	pericarp	[41]
*Citrus reticulata* Blanco	fruit	[41]
*Galdenia jasminoides* Ellis	whole plant	[41]
*Viola philipica* Cav.	stem	[41]
*Uncaria rhynchophylla*	leaf	[41]
*Babreum coscluea*	leaf	[41]
*Morus alba* L.	leaf	[48]
*Portulaca oleracea*	fruit	[49]
*Vitis vinifera* L.	leaf	[49]
*Salvia officinalis* L.	leaf	[49]

## References

[1] Dubois M., van den Broeck L., Inzé D. (2018). The pivotal role of ethylene in plant growth. Trends Plant Sci..

[2] Domingos P., Prado A.M., Wong A., Gehring C., Feijo J.A. (2015). Nitric oxide: A multitasked signaling gas in plants. Mol. Plant.

[3] Li Z.G., Min X., Zhou Z.H. (2016). Hydrogen sulfide: A signal molecule in plant cross-adaptation. Front. Plant Sci..

[4] Gilroy S., Białasek M., Suzuki N., Górecka M., Devireddy A.R., Karpiński S., Mittler R. (2016). ROS, calcium, and electric signals: Key mediators of rapid systemic signaling in plants. Plant Physiol..

[5] Martínez-Medina A., Fernandez I., Lok G.B., Pozo M.J., Pieterse C.M., van Wees S. (2017). Shifting from priming of salicylic acid-to jasmonic acid-regulated defences by Trichoderma protects tomato against the root knot nematode *Meloidogyne incognita*. New Phytol..

[6] Li W., Yamaguchi S., Khan M.A., An P., Liu X., Tran L.S.P. (2016). Roles of gibberellins and abscisic acid in regulating germination of *Suaeda salsa* dimorphic seeds under salt stress. Front. Plant Sci..

[7] Yoshida T., Mogami J., Yamaguchi-Shinozaki K. (2015). Omics approaches toward defining the comprehensive abscisic acid signaling network in plants. Plant Cell Physiol..

[8] Lerner A.B., Case J.D., Takahashi Y., Lee T.H., Mori W. (1958). Isolation of melatonin, a pineal factor that lightens melanocytes. J. Am. Chem. Soc..

[9] Pieri C., Marra M., Moroni F., Recchioni R., Marcheselli F. (1994). Melatonin: A peroxyl radical scavenger more effective than vitamin E. Life Sci..

[10] Rodriguez C., Mayo J.C., Sainz R.M., Antolin I., Herrera F., Martin V., Reiter R.J. (2004). Regulation of antioxidant enzymes: A significant role for melatonin. J. Pineal Res..

[11] Brainard G.C., Hanifin J.P., Greeson J.M., Byrne B., Glickman G., Gerner E., Rollag M.D. (2001). Action spectrum for melatonin regulation in humans: Evidence for a novel circadian photoreceptor. J. Neurosci..

[12] Brugger P., Marktl W., Herold M. (1995). Impaired nocturnal secretion of melatonin in coronary heart disease. Lancet.

[13] Cardinali D.P., Brusco L.I., Liberczuk C., Furio A.M. (2002). The use of melatonin in Alzheimer’s disease. Neuroendocrinol. Lett..

[14] Dollins A.B., Zhdanova I.V., Wurtman R.J., Lynch H.J., Deng M.H. (1994). Effect of inducing nocturnal serum melatonin concentrations in daytime on sleep, mood, body temperature, and performance. Proc. Natl. Acad. Sci. USA.

[15] Hattori A., Migitaka H., Iigo M., Itoh M., Yamamoto K., Ohtani-Kaneko R., Hara M., Suzuki T., Reiter R.J. (1995). Identification of melatonin in plants and its effects on plasma melatonin levels and binding to melatonin receptors in vertebrates. Biochem. Mol. Boil. Int..

[16] Dubbels R., Reiter R.J., Klenke E., Goebel A., Schnakenberg E., Ehlers C., Schiwara H.W., Schloot W. (1995). Melatonin in edible plants identified by radioimmunoassay and by high performance liquid chromatography-mass spectrometry. J. Pineal Res..

[17] Bajwa V.S., Shukla M.R., Sherif S.M., Murch S.J., Saxena P.K. (2014). Role of melatonin in alleviating cold stress in *Arabidopsis thaliana*. J. Pineal Res..

[18] Li C., Wang P., Wei Z., Liang D., Liu C., Yin L., Jia D., Fu M., Ma F. (2012). The mitigation effects of exogenous melatonin on salinity-induced stress in *Malus hupehensis*. J. Pineal Res..

[19] Wang L., Feng C., Zheng X., Guo Y., Zhou F., Shan D., Liu X., Kong J. (2017). Plant mitochondria synthesize melatonin and enhance the tolerance of plants to drought stress. J. Pineal Res..

[20] Martinez V., Nieves-Cordones M., Lopez-Delacalle M., Rodenas R., Mestre T.C., Garcia-Sanchez F., Rubio F., Nortes P.A., Mittler R., Rivero R.M. (2018). Tolerance to stress combination in tomato plants: New insights in the protective role of melatonin. Molecules.

[21] Kobylińska A., Borek S., Posmyk M.M. (2018). Melatonin redirects carbohydrates metabolism during sugar starvation in plant cells. J. Pineal Res..

[22] Hernandez-Ruiz J., Cano A., Arnao M.B. (2004). Melatonin: A growth-stimulating compound present in lupin tissues. Planta.

[23] Wang P., Yin L., Liang D., Li C., Ma F., Yue Z. (2012). Delayed senescence of apple leaves by exogenous melatonin treatment: Toward regulating the ascorbate-glutathione cycle. J. Pineal Res..

[24] Arnao M.B., Hernández-Ruiz J. (2009). Protective effect of melatonin against chlorophyll degradation during the senescence of barley leaves. J. Pineal Res..

[25] Murch S.J., Campbell S.S., Saxena P.K. (2001). The role of serotonin and melatonin in plant morphogenesis: Regulation of auxin-induced root organogenesis in in vitro-cultured explants of St. John’s wort (*Hypericum perforatum* L.). In Vitro Cell. Dev. Biol.-Plant.

[26] Kolář J., Johnson C.H., Macháčková I. (2003). Exogenously applied melatonin (*N*-acetyl-5-methoxytryptamine) affects flowering of the short-day plant *Chenopodium rubrum*. Physiol. Plant.

[27] Byeon Y., Park S., Kim Y.S., Park D.H., Lee S., Back K. (2012). Light-regulated melatonin biosynthesis in rice during the senescence process in detached leaves. J. Pineal Res..

[28] Van Tassel D.L., Roberts N., Lewy A., O’neill S.D. (2001). Melatonin in plant organs. J. Pineal Res..

[29] Okazaki M., Ezura H. (2009). Profiling of melatonin in the model tomato (*Solanum lycopersicum* L.) cultivar Micro-Tom. J. Pineal Res..

[30] Shi H., Reiter R.J., Tan D.X., Chan Z. (2015). INDOLE-3-ACETIC ACID INDUCIBLE 17 positively modulates natural leaf senescence through melatonin-mediated pathway in Arabidopsis. J. Pineal Res..

[31] Afreen F., Zobayed S.M.A., Kozai T. (2006). Melatonin in Glycyrrhiza uralensis: Response of plant roots to spectral quality of light and UV-B radiation. J. Pineal Res..

[32] Arnao M.B., Hernández-Ruiz J. (2013). Growth conditions determine different melatonin levels in *Lupinus albus* L.. J. Pineal Res..

[33] Byeon Y., Back K. (2014). Melatonin synthesis in rice seedlings in vivo is enhanced at high temperatures and under dark conditions due to increased serotonin *N*-acetyltransferase and *N*-acetylserotonin methyltransferase activities. J. Pineal Res..

[34] Posmyk M.M., Janas K.M. (2009). Melatonin in plants. Acta Physiol. Plant.

[35] Murch S.J., KrishnaRaj S., Saxena P.K. (2000). Tryptophan is a precursor for melatonin and serotonin biosynthesis in in vitro regenerated St. John’s wort (*Hypericum perforatum* L. cv. Anthos) plants. Plant Cell Rep..

[36] Zuo B., Zheng X., He P., Wang L., Lei Q., Feng C., Zhou J., Li Q., Han Z., Kong J. (2014). Overexpression of *MzASMT* improves melatonin production and enhances drought tolerance in transgenic *Arabidopsis thaliana* plants. J. Pineal Res..

[37] Tan D.X., Hardeland R., Back K., Manchester L.C., Alatorre-Jimenez M.A., Reiter R.J. (2016). On the significance of an alternate pathway of melatonin synthesis via 5-methoxytryptamine: Comparisons across species. J. Pineal Res..

[38] Choi G.H., Lee H.Y., Back K. (2017). Chloroplast overexpression of rice caffeic acid *O*-methyltransferase increases melatonin production in chloroplasts via the 5-methoxytryptamine pathway in transgenic rice plants. J. Pineal Res..

[39] Lee K., Lee H.Y., Back K. (2018). Rice histone deacetylase 10 and Arabidopsis histone deacetylase 14 genes encode *N*-acetylserotonin deacetylase, which catalyzes conversion of *N*-acetylserotonin into serotonin, a reverse reaction for melatonin biosynthesis in plants. J. Pineal Res..

[40] Badria F.A. (2002). Melatonin, serotonin, and tryptamine in some Egyptian food and medicinal plants. J. Med. Food.

[41] Chen G., Huo Y., Tan D.X., Liang Z., Zhang W., Zhang Y. (2003). Melatonin in Chinese medicinal herbs. Life Sci..

[42] Iriti M., Varoni E.M., Vitalini S. (2010). Melatonin in traditional Mediterranean diets. J. Pineal Res..

[43] Hernández-Ruiz J., Arnao M.B. (2008). Distribution of melatonin in different zones of lupin and barley plants at different ages in the presence and absence of light. J. Agric. Food Chem..

[44] Arnao M.B., Hernández-Ruiz J. (2013). Growth conditions influence the melatonin content of tomato plants. Food Chem..

[45] Boccalandro H.E., González C.V., Wunderlin D.A., Silva M.F. (2011). Melatonin levels, determined by LC-ESI-MS/MS, fluctuate during the day/night cycle in *Vitis vinifera* cv Malbec: Evidence of its antioxidant role in fruits. J. Pineal Res..

[46] Arnao M.B., Hernández-Ruiz J. (2009). Chemical stress by different agents affects the melatonin content of barley roots. J. Pineal Res..

[47] Okazaki M., Higuchi K., Aouini A., Ezura H. (2010). Lowering intercellular melatonin levels by transgenic analysis of indoleamine 2,3-dioxygenase from rice in tomato plants. J. Pineal Res..

[48] Simopoulos A.P., Tan D.X., Manchester L.C., Reiter R.J. (2005). Purslane: A plant source of omega-3 fatty acids and melatonin. J. Pineal Res..

[49] Stege P.W., Sombra L.L., Messina G., Martinez L.D., Silva M.F. (2010). Determination of melatonin in wine and plant extracts by capillary electrochromatography with immobilized carboxylic multi-walled carbon nanotubes as stationary phase. Electrophoresis.

[50] Wang P., Sun X., Li C., Wei Z., Liang D., Ma F. (2013). Long-term exogenous application of melatonin delays drought-induced leaf senescence in apple. J. Pineal Res..

[51] Li C., Tan D.X., Liang D., Chang C., Jia D., Ma F. (2014). Melatonin mediates the regulation of ABA metabolism, free-radical scavenging, and stomatal behaviour in two *Malus* species under drought stress. J. Exp. Bot..

[52] Meng J.F., Xu T.F., Wang Z.Z., Fang Y.L., Xi Z.M., Zhang Z.W. (2014). The ameliorative effects of exogenous melatonin on grape cuttings under water-deficient stress: Antioxidant metabolites, leaf anatomy, and chloroplast morphology. J. Pineal Res..

[53] Zhang N., Zhao B., Zhang H.J., Weeda S., Yang C., Yang Z.C., Ren S., Guo Y.D. (2013). Melatonin promotes water-stress tolerance, lateral root formation, and seed germination in cucumber (*Cucumis sativus* L.). J. Pineal Res..

[54] Lei X.Y., Zhu R.Y., Zhang G.Y., Dai Y.R. (2004). Attenuation of cold-induced apoptosis by exogenous melatonin in carrot suspension cells: The possible involvement of polyamines. J. Pineal Res..

[55] Posmyk M.M., Bałabusta M., Wieczorek M., Sliwinska E., Janas K.M. (2009). Melatonin applied to cucumber (*Cucumis sativus* L.) seeds improves germination during chilling stress. J. Pineal Res..

[56] Zhao Y., Qi L.W., Wang W.M., Saxena P.K., Liu C.Z. (2011). Melatonin improves the survival of cryopreserved callus of *Rhodiola crenulata*. J. Pineal Res..

[57] Uchendu E.E., Shukla M.R., Reed B.M., Saxena P.K. (2013). Melatonin enhances the recovery of cryopreserved shoot tips of American elm (*Ulmus americana* L.). J. Pineal Res..

[58] Turk H., Erdal S., Genisel M., Atici O., Demir Y., Yanmis D. (2014). The regulatory effect of melatonin on physiological, biochemical and molecular parameters in cold-stressed wheat seedlings. Plant Growth Regul..

[59] Shi H., Jiang C., Ye T., Tan D.X., Reiter R.J., Zhang H., Liu R., Chan Z. (2015). Comparative physiological, metabolomic, and transcriptomic analyse sreveal mechanisms of improved abiotic stress resistance in bermudagrass [*Cynodon dactylon* (L). Pers.] by exogenous melatonin. J. Exp. Bot..

[60] Fan J., Hu Z., Xie Y., Chan Z., Chen K., Amombo E., Chen L., Fu J. (2015). Alleviation of cold damage to photosystem II and metabolisms by melatonin in Bermudagrass. Front. Plant Sci..

[61] Hu Z., Fan J., Xie Y., Amombo E., Liu A., Gitau M.M., Khaldun A.B.M., Chen L., Fu J. (2016). Comparative photosynthetic and metabolic analyses reveal mechanism of improved cold stress tolerance in bermudagrass by exogenous melatonin. Plant Physiol. Biochem..

[62] Tal O., Haim A., Harel O., Gerchman Y. (2011). Melatonin as an antioxidant and its semi-lunar rhythm in green macroalga *Ulva* sp.. J. Exp. Bot..

[63] Tiryaki I., Keles H. (2012). Reversal of the inhibitory effect of light and high temperature on germination of *Phacelia tanacetifolia* seeds by melatonin. J. Pineal Res..

[64] Hernández I.G., Gomez F.J.V., Cerutti S., Arana M.V., Silva M.F. (2015). Melatoninin *Arabidopsis thaliana* acts as plant growth regulator at low concentrations and preserves seed viability at high concentrations. Plant Physiol. Biochem..

[65] Parida A.K., Das A.B. (2005). Salt tolerance and salinity effects on plants: A review. Ecotoxicol. Environ. Saf..

[66] Zhang N., Zhang H.J., Zhao B., Sun Q.Q., Cao Y.Y., Li R., Wu X.X., Weeda S., Li L., Ren S. (2014). The RNA-seq approach to discriminate gene expression profiles in response to melatonin on cucumber lateral root formation. J. Pineal Res..

[67] Wei W., Li Q.T., Chu Y.N., Reiter R.J., Yu X.M., Zhu D.H., Zhang W.K., Ma B., Lin Q., Zhang J.S. (2015). Melatonin enhances plant growth and abiotic stress tolerance in soybean plants. J. Exp. Bot..

[68] Zhu J.K. (2003). Regulation of ion homeostasis under salt stress. Curr. Opin. Plant Boil..

[69] Hamayun M., Khan S.A., Khan A.L., Shin J.H., Ahmad B., Shin D.H., Lee I.J. (2010). Exogenous gibberellic acid reprograms soybean to higher growth and salt stress tolerance. J. Agric. Food Chem..

[70] Zhang H.J., Zhang N., Yang R.C., Wang L., Sun Q.Q., Li D.B., Cao Y.Y., Weeda S., Zhao B., Ren S. (2014). Melatonin promotes seed germination under high salinity by regulating antioxidant systems, ABA and GA_4_ interaction in cucumber (*Cucumis sativus* L.). J. Pineal Res..

[71] Tan D.X., Manchester L.C., Helton P., Reiter R.J. (2007). Phytoremediative capacity of plants enriched with melatonin. Plant Signal. Behav..

[72] Posmyk M.M., Kuran H., Marciniak K., Janas K.M. (2008). Presowing seed treatment with melatonin protects red cabbage seedlings against toxic copper ion concentrations. J. Pineal Res..

[73] Hasan M., Ahammed G.J., Yin L., Shi K., Xia X., Zhou Y., Yu J., Zhou J. (2015). Melatonin mitigates cadmium phytotoxicity through modulation of phytochelatins biosynthesis, vacuolar sequestration, and antioxidant potential in *Solanum lycopersicum* L.. Front. Plant Sci..

[74] Kang K., Lee K., Park S., Byeon Y., Back K. (2013). Molecular cloning of rice serotonin *N*-acetyltransferase, the penultimate gene in plant melatonin biosynthesis. J. Pineal Res..

[75] Shi H., Chan Z. (2014). The cysteine2/histidine2-type transcription factor ZINC FINGER OF ARABIDOPSIS THALIANA 6-activated C-REPEAT-BINDING FACTOR pathway is essential for melatonin-mediated freezing stress resistance in Arabidopsis. J. Pineal Res..

[76] Wei Y., Liu G., Chang Y., Lin D., Reiter R.J., He C., Shi H. (2018). Melatonin biosynthesis enzymes recruit WRKY transcription factors to regulate melatonin accumulation and transcriptional activity on W-box in cassava. J. Pineal Res..

[77] Shi H., Tan D.X., Reiter R.J., Ye T., Yang F., Chan Z. (2015). Melatonin induces class A1 heat-shock factors (HSFA1s) and their possible involvement of thermotolerance in Arabidopsis. J. Pineal Res..

[78] Hernández-Ruiz J., Cano A., Arnao M.B. (2005). Melatonin acts as a growth-stimulating compound in some monocot species. J. Pineal Res..

[79] Arnao M.B., Hernández-Ruiz J. (2007). Melatonin promotes adventitious-and lateral root regeneration in etiolated hypocotyls of *Lupinus albus* L.. J. Pineal Res..

[80] Chen L., Fan J., Hu Z., Huang X., Amombo E., Liu A., Bi A., Chen K., Xie Y., Fu J. (2017). Melatonin is involved in regulation of Bermudagrass growth and development and response to low K^+^ stress. Front. Plant Sci..

[81] Kolář J., Macháčková I., Eder J., Prinsen E., van Dongen W., van Onckelen H., Illnerová H. (1997). Melatonin: Occurrence and daily rhythm in *Chenopodium rubrum*. Phytochemistry.

[82] Hernández-Ruiz J., Arnao M.B. (2008). Melatonin stimulates the expansion of etiolated lupin cotyledons. Plant Growth Regul..

[83] Yin L., Wang P., Li M., Ke X., Li C., Liang D., Wu S., Ma X., Li C., Zou Y. (2013). Exogenous melatonin improves *Malus* resistance to *Marssonina* apple blotch. J. Pineal Res..

[84] Lee H.Y., Byeon Y., Back K. (2014). Melatonin as a signal molecule triggering defense responses against pathogen attack in Arabidopsis and tobacco. J. Pineal Res..

[85] Shi H., Chen Y., Tan D.X., Reiter R.J., Chan Z., He C. (2015). Melatonin induces nitric oxide and the potential mechanisms relate to innate immunity against bacterial pathogen infection in Arabidopsis. J. Pineal Res..

[86] Wei Y., Hu W., Wang Q., Zeng H., Li X., Yan Y., Reiter R.J., He C., Shi H. (2017). Identification, transcriptional and functional analysis of heat-shock protein 90 s in banana (*Musa acuminata* L.) highlight their novel role in melatonin-mediated plant response to *Fusarium wilt*. J. Pineal Res..

[87] Manchester L.C., Tan D.X., Reiter R.J., Park W., Monis K., Qi W. (2000). High levels of melatonin in the seeds of edible plants. Possible function in germ tissue protection. Life Sci..

[88] Tan D.X., Hardeland R., Manchester L.C., Korkmaz A., Ma S., Rosales-Corral S., Reiter R.J. (2012). Functional roles of melatonin in plants, and perspectives in nutritional and agricultural science. J. Exp. Bot..

[89] Manchester L.C., Coto-Montes A., Boga J.A., Andersen L.P.H., Zhou Z., Galano A., Vriend J., Tan D.X., Reiter R.J. (2015). Melatonin: An ancient molecule that makes oxygen metabolically tolerable. J. Pineal Res..

[90] Lee H.Y., Back K. (2017). Melatonin is required for H_2_O_2_-and NO-mediated defense signaling through MAPKKK3 and OXI1 in Arabidopsis thaliana. J. Pineal Res..

[91] Liang C., Zheng G., Li W., Wang Y., Hu B., Wang H., Wu H., Qian Y., Zhu X.G., Tan D.X. (2015). Melatonin delays leaf senescence and enhances salt stress tolerance in rice. J. Pineal Res..

